# Modeling Biological Systems: Trends in Biosystem Identification and Biosignal Coupling

**DOI:** 10.1055/s-0046-1816599

**Published:** 2026-11-03

**Authors:** Kyriaki Kostoglou, Gernot R. Müller-Putz

**Affiliations:** 1 Institute of Neural Engineering, Graz University of TechnologyGrazAustria; 2 BioTechMedGrazAustria

**Keywords:** Physiological Modeling, Biosignal Coupling, Nonlinear Dynamics, Time-varying systems, Machine Learning

## Abstract

**Objectives:**

Biosystem identification is a multidisciplinary approach to modeling and understanding the dynamic behavior of biological systems through the analysis of physiological signals. Complementary to biosystem identification, biosignal coupling quantifies interactions between different physiological signals, revealing important dependencies in systems like neuro-cardio-respiratory interactions and brain network dynamics. This commentary paper explores current trends and emerging methodologies in these areas.

**Methods:**

We present a survey of advanced time-varying and nonlinear modeling approaches, explore sophisticated techniques for biosignal coupling, and highlight the integration of machine learning and deep learning frameworks within biosystem identification. We also discuss applications in real-time monitoring, personalized medicine, and clinical diagnostics, illustrating the potential of these methodologies in both biomedical research and healthcare.

**Conclusion:**

Despite significant advancements in the field, the ability to model complex physiological systems and extract metrics with direct clinical relevance remains an ongoing challenge and area of active research. Continued progress in computational methods and data acquisition technologies hold great promise for broadening the scope and enhancing the impact of biosystem identification.

## 1. Introduction

Biosystem identification, a rapidly advancing field in biomedical engineering, focuses on developing mathematical models to analyze and understand dynamic interactions within biological systems [ 1 , 2 ]. By utilizing biomedical signals — measurable representations of the body’s physiological processes such as those of the cardiovascular, nervous, muscular and respiratory systems — biosystem identification captures the complex relationships between physiological inputs and outputs, offering a deeper understanding of the underlying system behavior and functionality. For example, in the cardiovascular system, cerebral autoregulation, a mechanism that maintains consistent blood flow to the brain despite fluctuations in arterial pressure, is characterized by the relationship between arterial blood pressure (input) and cerebral blood velocity (output) [ 3 ]. When this mechanism is impaired, such as in stroke or traumatic brain injury, biosystem identification reveals disruptions in the input-output relationship, highlighting deviations from normal physiological function [ 4 – 7 ] and indicating potential risks to the patient’s health.

Another critical cardiovascular mechanism, the baroreflex, is often modeled by analyzing the relationship between blood pressure (input) and heart rate (output) [ 8 ]. Baroreflex operates as a feedback control system, where specialized sensors called baroreceptors detect changes in blood pressure and trigger adjustments in heart rate and blood vessel tone. This process involves not only peripheral mechanisms [ 9 ] but also direct connections to the brain. Baroreceptors transmit signals to the brainstem, where the information is processed and used to modulate autonomic responses [ 10 ]. Thus, the baroreflex can also be studied by examining the relationships between blood pressure (input) and brain activity (intermediate output), and then brain activity (intermediate input) and heart rate (output) [ 11 , 12 ], emphasizing the brain’s role in mediating the dynamic interplay between cardiovascular signals and autonomic responses. Impairments in the baroreflex can lead to conditions such as orthostatic hypotension or hypertension [ 13 ], underscoring the importance of detecting these disruptions to better understand and manage the associated physiological and clinical implications. In neuroscience, biosystem identification extends to studying the function of an individual neuron based on the relationship between local field potentials (LFP)—representing the collective activity of a population of neurons within a localized brain region—and the neuron’s spiking response to this activity [ 14 – 17 ]. This connection provides valuable insights into how neurons process information and contribute to the overall dynamics of the network. Impairments in this relationship, such as in epilepsy or neurodegenerative diseases, are detectable through identification, revealing abnormal neural behaviors.

A closely related field, biosignal coupling, focuses specifically on quantifying and analyzing the interactions between different physiological signals, such as heart rate and respiration or neural oscillations across brain regions. While biosignal coupling is often a subset of biosystem identification, biosignal coupling emphasizes the study of interdependencies between signals, rather than direct input-output modeling. Neuro-cardio-respiratory interactions in epilepsy, for example, are a key area of research, as seizures often disrupt autonomic regulation. Such disruptions can impair the dynamic balance between the brain, heart, and lungs, increasing the risk of sudden unexpected death in epilepsy [ 18 , 19 ]. Measuring these interactions help identify biomarkers for risk assessment and early intervention. Similarly, brain-respiration coupling, which involves the influence of respiratory rhythms on neural oscillations, play a key role in supporting various cognitive and emotional processes [ 20 ]. This coupling is often impaired in neuropsychiatric disorders such as anxiety [ 11 , 21 ]. Biosignal coupling is also widely used to investigate brain networks using electroencephalography (EEG) or electrocorticography (ECoG). In clinical research, disruptions in EEG/ECoG coupling serves as a biomarker for neurological conditions such as epilepsy [ 22 ], schizophrenia [ 23 ], or Alzheimer’s disease [ 24 ].

## 2. Methods and Results

We focus here on current trends in Biosignal and Imaging Informatics.

### 2.1. Trends in Biosystem Identification and Biosignal Coupling

Historically, biosystem identification has utilized both time domain and spectral domain techniques to characterize the interactions between physiological inputs and outputs. These two approaches are complementary, providing distinct yet interconnected perspectives on system behavior. Time-domain methods operate directly in the temporal domain, capturing system dynamics through impulse or step responses [ 25 ]. Examples of time-domain techniques are linear time-invariant (LTI) models, such as finite impulse response (FIR) systems—either based on direct estimation or basis function expansions— [ 2 , 25 ] and infinite impulse response (IIR) systems, such as autoregressive models with exogenous inputs (ARX) and their variants [ 26 ]. These techniques have found application in a wide range of physiological contexts, such as modeling of insulin-glucose dynamics [ 27 ], cerebral autoregulation [ 4 – 7 , 28 , 29 ], fluid balance changes in intensive care patients [ 30 ], and even ion channel dynamics on a microscopic level [ 31 ]. Frequency-domain identification, in contrast, operates directly in the spectral domain, analysing how systems respond to inputs at various frequencies. Common techniques include transfer function analysis, which characterizes the frequency response of a system and has been widely applied in the assessment of cerebral autoregulation [ 3 ]. Time-domain approaches are often more flexible in model formulation, as they more easily incorporate structured noise and multiple physiological inputs without implying, however, that similar capabilities are entirely absent in frequency-domain methods. Another prominent category is biophysical and compartmental models, which use mathematical representations of physiological systems grounded in known physical and biological principles. These models can operate in either the temporal or frequency domain, depending on the application. Biophysical models provide insights into cerebral blood flow dynamics [ 32 ] and cancer growth [ 33 ], while in neural modeling, multi-compartmental models facilitate the study of signal propagation and processing within neurons [ 34 ] and astrocytes [ 35 ].

In biosignal coupling, various methods have also been developed to quantify interdependencies between physiological signals. Coherence analysis [ 36 ] measures synchronization in the frequency-domain, capturing how signals oscillate together at different frequencies, while cross-correlation evaluates the temporal alignment and the strength of association between signals in the time domain [ 36 ]. Multivariate autoregressive (MVAR) models [ 37 ] extend traditional approaches by modeling dependencies between multiple physiological signals. These models enable the derivation of advanced measures such as partial directed coherence and directed coherence [ 38 ] describing directional coupling in the spectral domain. Partial directed coherence isolates direct influences, meaning it identifies the impact of one signal on another without the interference of intermediate signals. Directed coherence, on the other hand, captures both direct and indirect couplings, meaning it reflects both the immediate influence of one signal on another and connections that arise through intermediate links. Granger causality [ 39 ], on the other hand, identifies directional influences between signals in the temporal domain. Unlike coherence, which only detects shared oscillatory patterns, Granger causality determines whether one signal contains predictive information about another, offering a way to infer effective connectivity within physiological networks. In the past, these methods have seen success in various contexts, including analyzing corticomuscular [ 40 ] and corticospinal [ 41 ] coupling, heart rate and respiratory synchronization [ 42 ], hemodynamic interactions [ 43 ], effective brain connectivity [ 44 – 46 ] and feature extraction for brain-computer interface control [ 47 , 48 ].

A prominent trend in biosystem identification is the increasing focus on time-varying and non-linear estimation, driven by the dynamic and complex behavior of biological systems. Unlike traditional models that assume unchanging system behavior, time-varying methods are specifically designed to account for variations over time. These variations may stem from inherent system dynamics, shifting external conditions, or unobservable factors. One widely used time-varying identification approach involves the quasistationary sliding window method, which divides the data into overlapping windows of fixed length. In each window, the system is assumed to remain stationary, allowing for the detection and tracking of temporal changes in the system’s behavior. However, the reliance on fixed window lengths may limit responsiveness to rapid shifts in system behavior when windows are long or introduce estimation noise when windows are too short. A more refined approach involves recursive methods (e.g., recursive least squares, Kalman filtering, particle filters) which are iterative algorithms that continuously update the model as new data is acquired [ 26 , 49 ], offering greater adaptability. Recursive estimation facilitates the tracking of cardiovascular responses to acute exercise [ 43 ], cerebral autoregulation changes during normal [ 50 ], hypercapnic conditions [ 51 ] and vasovagal syncope [ 52 ], as well as EEG dynamic variations for brain computer interface use [ 53 – 55 ]. These adaptive modeling capabilities not only enhance the accuracy of biosystem representations but also facilitate the development of real-time monitoring systems. Alternatively, basis function expansions [ 56 ] rely on a combination of mathematical functions to represent a system’s evolving behavior over time. While they are particularly effective for capturing smooth temporal changes—such as the dynamic stiffness of the elbow described by the varying relationship between joint position and force [ 57 ]—they are typically applied offline, making them less suitable for applications requiring real-time adaptation.

Another powerful approach is linear parameter varying techniques [ 58 ], which provide a structured framework for modeling systems whose behavior varies in relation to an external variable. These models have been used to explore the time-varying relationship between heart rate and respiration during respiratory sinus arrhythmia [ 59 ], with respiration serving as the external factor that modulates heart rate dynamics. On the other hand, nonlinear estimation methods overcome the limitations of linear models by capturing complex, nonlinear interactions that are inherent in many biological processes. Volterra series, for example, extend linear models by incorporating higher-order interactions. Nonlinear Laguerre-Volterra models effectively describe the nonlinear dynamics of hippocampal neuronal networks [ 60 ] or hemodynamic mechanisms [ 61 ]. While these models are flexible and interpretable, their computational cost increases with the degree of nonlinearity. Block-structured models such as the Hammerstein-Wiener model, combine nonlinear and linear components in series to capture complex dynamics. They are especially appropriate when the system structure is partially known and includes separable linear and nonlinear components. These models have provided useful information regarding the mechanical properties of human joints and stretch flex dynamics [ 62 ], as well as heart rate regulation [ 63 ]. Nonlinear autoregressive models with exogenous inputs (NARX/NARMAX) represent the output of a system as a nonlinear function of past input and output values [ 64 ]. They have been applied to model brain responses to wrist perturbations [ 65 ], providing a deeper understanding of sensorimotor processes, as well as to study thalamocortical interactions underlying essential tremor [ 66 ]. However, these models are generally less interpretable, necessitating careful model design and validation.

To address the complexities of biosignal coupling, a variety of advanced techniques have recently emerged. Similarly to biosystem identification, recursive and quasistationary approaches serve as foundational methods for analyzing dynamic interactions over time. For example, these methods have been employed in studies investigating corticomuscular coherence [ 67 ], time-varying information measures related to heart-brain coupling [ 68 ] and event-related synchronization of different brain regions during motor tasks [ 69 , 70 ]. In Figure 1 , we demonstrate the application of such techniques in the study of corticomuscular coupling. The figure highlights the use of MVAR-based directed coherence to capture the time-varying directional influence of cortical activity, measured via EEG, on muscle activation, recorded via electromyography (EMG) during the execution of brief hand movements. By applying a sliding window approach, the method tracks dynamic changes in coupling across the spectral domain, providing insight into both preparatory and post-movement neural processes. The figure also integrates event-related spectral analysis offering a reference for well-established cortical activity patterns during movement initiation and execution, which in turn helps to interpret connectivity dynamics across different movement phases. The results highlight distinct pre-movement and post-movement coupling phases, where low-frequency coherence precedes movement onset, and beta-band coherence emerges after movement. This example was selected to highlight the adaptive nature of biosignal coupling, which continuously evolves in response to physiological conditions, external influences, and internal regulatory mechanisms. Figure 1 captures this evolving interaction showing how cortical connectivity fluctuates throughout movement execution and recovery. By associating connectivity patterns with distinct motor phases, this approach offers a clearer understanding of movement-related neural processes.

**Figure 1:**
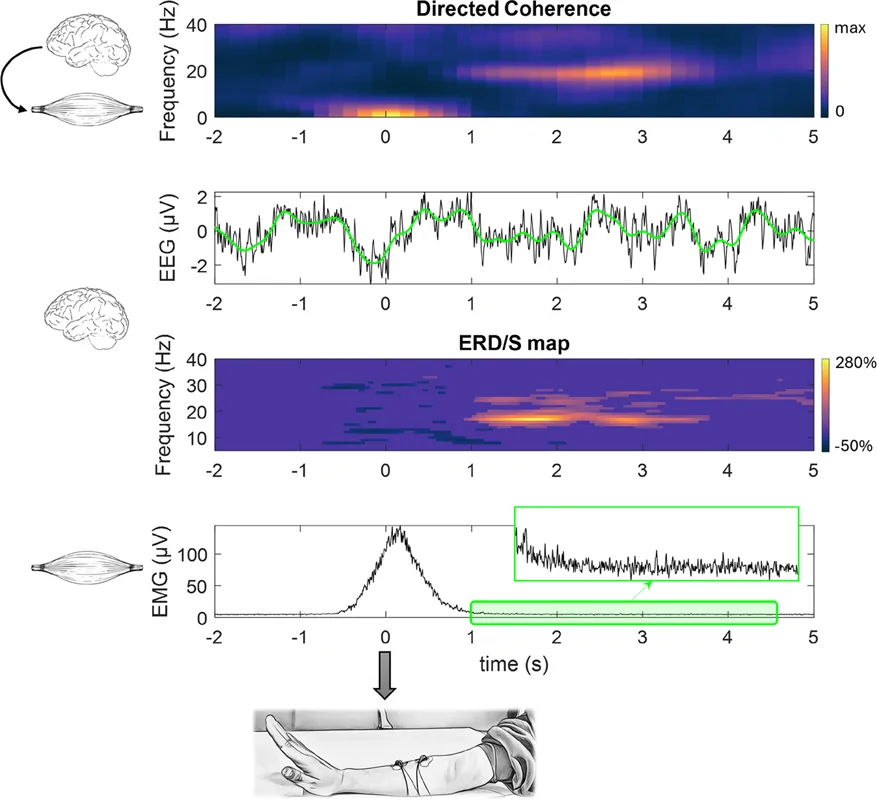
. Corticomuscular coupling – The top panel illustrates the time-varying directional influence of EEG on EMG in the spectral domain, as measured by MVAR-based directed coherence (top panel) using a sliding window approach. A healthy participant performed brief hand movements, specifically simultaneous wrist and finger extension, with the right hand [ 88 ].

Another key method for analyzing nonstationary signals is wavelet coherence [ 71 ], which measures the local correlation between two signals in both time and frequency domain, revealing phenomena such as the impact of muscle fatigue on corticomuscular coupling [ 72 ]. Phase synchronization measures the alignment of the phases of oscillatory signals and plays a crucial role in neuroscience for studying neural synchronization, including its involvement in memory processes [ 73 ], motor imagination [ 74 ] and in sensorimotor brain-computer interface control for conditions like cerebral palsy [ 75 ] and spinal cord injury [ 76 ]. Cross-frequency coupling techniques that analyze interactions between different frequency components of signals provide insights into, for example, learning and memory [ 77 ], motor actions [ 78 ] and neural dynamics during the gait cycle [ 79 ] by analyzing brain activity. Bispectral analysis detects higher-order interactions and harmonics in coupled systems, proving particularly useful for analyzing epileptic EEG signals [ 80 ] or cardiac arrythmias [ 81 ]. Additionally, information theory-based methods provide a powerful framework for detecting complex relationships. For example, mutual information quantifies the amount of shared information between two signals and has been utilized to identify the role of synchronization between the cardiac and respiratory systems in emotion recognition [ 82 ]. Cross-entropy builds on this concept by comparing probability distributions between signals, quantifying how well one signal’s probability distribution predicts another. It has been particularly effective in cardiorespiratory coupling analysis, aiding in the differentiation of depression stages [ 83 ]. Transfer entropy, a non-parametric method, quantifies the directed information flow between signals. Unlike Granger causality, which assumes linear relationships, transfer entropy can capture nonlinear and time-lagged interactions. It has been instrumental in identifying causal relationships within cardiovascular and cardiorespiratory systems [ 84 ]. Graph theory has also become a powerful tool for studying biosignal coupling, representing signals or regions as nodes and their interactions as edges. Graph-based approaches provide a quantitative framework to study network properties, enabling researchers to map and analyze for example, large-scale connectivity patterns in neural [ 85 ] and vascular [ 86 ] systems. Finally, more advanced techniques also include coupled hidden Markov models, a type of statistical model where multiple hidden Markov processes are interconnected, with the hidden states of each process influencing each other, allowing for the modeling of complex dependencies between multiple signals [ 87 ].

Given the diversity of system identification and biosignal coupling techniques, choosing the appropriate approach depends on the specific objectives of the study. Time-varying models are particularly effective for tracking dynamic physiological processes but require greater computational resources and precise tuning. Nonlinear models are optimal at modeling complex interactions however, they can be computationally intensive, often require larger datasets for reliable estimation, and may lack interpretability. In contrast, linear models provide simplicity and efficiency but may fail to capture essential nonlinearities in biological systems. A clear understanding of the research goals, whether mechanistic insight, real-time tracking, or predictive accuracy, is essential for selecting the most suitable modeling strategy.

The experiment in Figure 1 was conducted at the Institute of Neural Engineering, Graz University of Technology, with the participant providing informed consent. Data acquisition and paradigm visualization were performed using the Medusa BCI Software [ 89 ]. The cue onset, marking the participant’s instruction to perform the movement [ 88 ], is indicated at t = 0 s. Data from 60 iterations of the movement task were used to estimate a single MVAR model at sliding window intervals around the cue onset. 60-channel EEG signals were recorded using a biosignal amplifier (Brain Products GmbH). EMG sensors were placed over the extensor pollicis longus muscle of the participant’s right forearm. The second panel depicts in black the average broadband EEG signal across all movement trials from a central channel in the left hemisphere (C1), where coherence was more pronounced contralateral to the hand movement in the motor cortex. EEG signals were bandpass-filtered in the [0.5–60] Hz frequency range (4th order Butterworth filter). Additionally, the EEG signal filtered in the [0.5 3] Hz frequency range is overlaid in green, capturing the well-known movement-related cortical potentials linked to the preparation and execution of voluntary movements [ 90 ]. The third panel depicts statistically significant Event-Related Desynchronization/Synchronization (ERD/S) phenomena, illustrating changes in the spectral power of the EEG signals (in C1) relative to a baseline period ([-2 -1] sec). The ERD/S map highlights frequency-specific decreases (ERD) and increases (ERS) in power, which are temporally and spatially linked to the movement trials [ 91 ]. These changes are computed across all trials and are represented as a time-frequency plot, with ERD observed in the alpha (8–12 Hz) and beta (13–30 Hz) bands during movement preparation and execution, while ERS is prominent in the post-movement period, particularly in the beta band, reflecting motor cortical reorganization and recovery processes. Statistical significance was evaluated using a bootstrap-based method. The fourth panel depicts the average high-pass filtered (30 Hz cut-off) and rectified EMG signal. The green inset highlights a zoomed-in view of the time interval [1–4.5] sec. Around the cue onset, an increase in directed coherence in the very low-frequency range is observed, representing preparatory cortical activity driving muscle activation. After the movement, a significant rise in directed coherence within the beta band (13–30 Hz) is observed, corresponding to the so-called post-movement beta synchronization. This phenomenon is associated with cortical reorganization and inhibition processes following voluntary movements, commonly observed in the motor cortex (also observed in the ERD/S map).

### 2.2. The Integration of Machine/Deep Learning

In today’s data-driven scientific landscape, the impact of machine learning (ML) and deep learning (DL) cannot, of course, be overlooked. Their integration has brought transformative advancements to system identification [ 92 – 94 ]. In light of this, we briefly outline some key trends that have emerged in this domain. Unlike traditional methods, such as Volterra series or kernel-based approaches, ML and DL models do not require pre-defined equations or assumptions. Instead, they can automatically learn intricate, nonlinear input-output relationships directly from data. Convolutional recurrent neural networks, long short-term memory networks, graph neural networks and various ML/DL architectures have recently been employed to model in biosystems [ 17 , 65 , 95 – 98 ].

One of the major advantages of ML and DL approaches is also their ability to integrate vast, heterogeneous datasets, facilitating generalization across diverse physiological conditions and populations. Furthermore, these techniques are particularly adept at handling high-dimensional data, such as those generated from advanced imaging modalities and multi-channel biosignals. Using approaches like dimensionality reduction, feature extraction, and hierarchical learning, DL models can identify meaningful patterns and relationships within this complex data while effectively filtering out noise and redundant information.

However, a significant drawback of ML and DL approaches is their reliance on large amounts of high-quality data for efficient training. Unlike traditional system identification methods that can work with relatively limited datasets, DL models often require extensive data to generalize well and avoid overfitting. This poses a major challenge, particularly in biomedical applications where data collection can be constrained by ethical considerations, participant limitations, high costs or even technical constraints. Additionally, while ML and DL approaches have significantly advanced the predictive capabilities of system identification, their lack of explainability remains a critical limitation. Explainability is not just a theoretical concern; it is essential for practical applications such as personalized medicine and real-time monitoring, where clinicians need to trust and understand model outputs. In fact, explainability has long been a central focus of system identification, as the field prioritizes not just accurate predictions but also a clear understanding of the underlying mechanisms and relationships within biological systems [ 2 , 99 ]. The recent development of digital twins [ 100 ], for example, showcases the importance of explainable models. Digital twins are virtual representations of biological systems that replicate their dynamic behavior in real time. However, for digital twins to be clinically useful, their foundational structure must be interpretable. For example, a digital twin of the cardiovascular system that models cerebral autoregulation or baroreflex mechanisms must provide clear, biologically relevant insights into how these systems respond to changes in blood pressure or treatment. To address this challenge, researchers are increasingly exploring explainable artificial intelligence (XAI) techniques within ML and DL [ 101 ]. XAI methods aim to make the decision-making processes of these models transparent and interpretable. Tools such as SHAP (SHapley Additive exPlanations) [ 102 ] and LIME (Local Interpretable Model-Agnostic Explanations) [ 103 ] can identify which input features contribute most to model predictions, providing an explanation of the physiological variables driving specific outcomes. Figure 2 illustrates how advanced modeling approaches that integrate system identification with machine learning can yield both interpretable representations of system dynamics and clinically meaningful results. In this figure, Spiking Laguerre-Volterra Networks (sLVN) [ 17 ] are used to model the relationship between continuous-valued inputs and binary outputs. Specifically, local field potentials (LFPs) serve as inputs, and neuronal spiking activity as output, based on data collected from microelectrode recordings of Parkinson’s disease patients undergoing subthalamic nucleus (STN) deep brain stimulation surgery. By combining feedforward neural networks with polynomial activation functions and Laguerre expansions at the input stage, the sLVN effectively captures nonlinear dynamic input-output relationships while providing insight into key LFP dynamic modes (also known as principal dynamic modes, PDMs). PDMs represent the dominant dynamic behaviors of a system and, in this context, serve as interpretable components that reveal how specific temporal features of the STN LFP signal contribute to spiking behavior. Importantly, the model’s ability to predict spiking activity also led to the identification of three distinct neuronal clusters, which were associated with motor improvement following deep brain stimulation surgery. This underscores the clinical relevance of the approach and its potential application in optimizing interventions such as deep brain stimulation.

**Figure 2:**
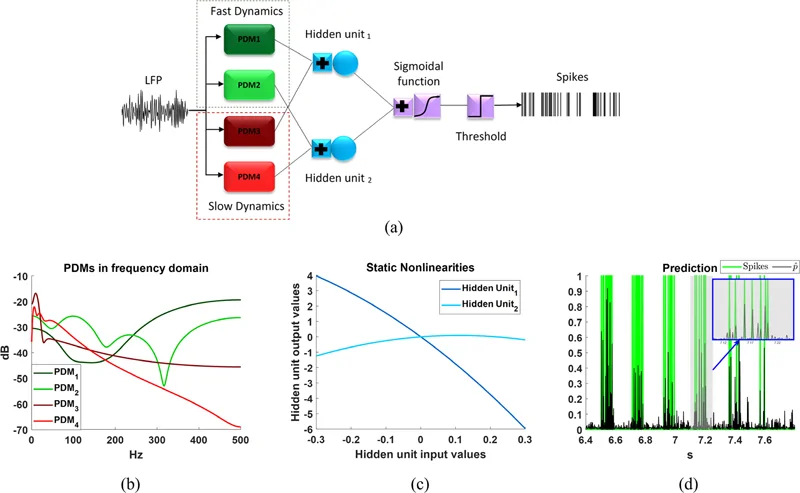
. Integrating ML in nonlinear system identification - (a) Spiking Laguerre-Volterra network (sLVN) developed to model the relationship between local field potentials (LFP) and neuronal spiking activity.

Figure 2 and sLVNs provide a form of explainable ML-driven system identification as these models offer intrinsic interpretability through their structured design and specifically through the PDMs. Furthermore, hybrid approaches such as physics-informed neural networks [ 104 – 106 ] integrate known physiological equations with ML/DL models, ensuring that predictions adhere to biological principles while maintaining flexibility. These techniques bridge the gap between traditional system identification and modern ML, preserving interpretability which is central to the field.

Data was collected from microelectrode recordings of Parkinson’s diseases patients undergoing subthalamic nucleus (STN) deep brain stimulation surgery. sLVNs combine feedforward neural networks with polynomial activation functions and Laguerre expansions at the input stage to model the nonlinear dynamic input–output relationship. This approach accounts also for the binary nature of the spiking signal, yielding as output the probability of firing a spike. The LFP is fed into multiple filters also known as principal dynamic modes (PDMs). The PDMs represent the dominant dynamic behaviors of a system. They are derived from input-output data using the Volterra series framework, combined with Laguerre functions to reduce dimensionality and computational complexity. In the Laguerre-Volterra framework the output of a system is expressed as a sum of multidimensional convolutions of the input signal with Volterra kernels (generalizations of impulse responses for nonlinear systems). The kernels are expanded into an orthogonal basis of Laguerre functions, simplifying the identification of the system. In (a) fast and slow system dynamics are captured by the green (PDM1 and PDM2) and red (PDM3 and PDM4) PDMs, respectively. Two hidden units with polynomial activation functions receive input from the PDMs. The output from the hidden units is transformed into probabilities of firing using a sigmoid function. An optimal threshold is finally applied to generate the spike signal. The bottom panel (i.e., (b)-(d)) presents the results of system identification from a STN recording: (b) Magnitude response of the PDMs, illustrating the dominant fast and slow system dynamics. (c) Static nonlinearities of the hidden units, showing the nonlinear transformations applied to the PDM outputs. (d) Comparison of actual spikes in green and sLVN predictions (as probabilities of firing) in black over a specified testing time window. The inset highlights a zoomed-in view of the time interval [7.10–7.25] sec. Modified with permission from [ 17 ] under the Creative Commons CC-BY license.

## 3. Conclusion

There is an extensive body of literature on biosystem identification, and it is important to acknowledge that this paper reflects a bias toward areas most familiar to the authors. Biosystem identification is a vast and interdisciplinary field, drawing contributions from diverse domains such as control theory, signal processing, neuroscience, computational biology, and clinical research. While significant progress has been made, there are still numerous challenges to overcome. Biological systems are complex, dynamic, and subject to unpredictable variations, making accurate modeling difficult. Furthermore, a central difficulty lies in extracting physiologically meaningful measures from these models. Translating mathematical representations into clinically interpretable biomarkers or decision-making tools requires the development of robust, well-validated metrics which is a complex task in itself and far from trivial. Looking ahead, however, the future of biosystem identification is profoundly exciting and filled with immense potential. Advances in computational methodologies, from machine learning to physics-informed models, and cutting-edge data acquisition technologies, such as multi-modal imaging and wearable sensors, will continue to push the boundaries of what can be modeled, analyzed, and understood in biological systems.
